# Changes in clinical and CT manifestations related to liver abscesses in patients with vs. without basic diabetes mellitus before and after CT-guided interventional therapy: An observational study

**DOI:** 10.1016/j.clinsp.2022.100164

**Published:** 2023-01-17

**Authors:** Yuxiang Chen, Xunfu Lai, Yuping Zhu, Mengting Wang, Yulin He

**Affiliations:** Department of Radiology, First Affiliated Hospital of Nanchang University; Jiangxi Medical College, Nanchang University, Nanchang, China

**Keywords:** Diabetes mellitus, Liver abscess, Interventional therapy, Tomography, X-ray computed, DM, Diabetes mellitus, LA, Liver abscess, CT, Computed tomography, PNA, Percutaneous needle aspiration, PCD, Percutaneous catheter drainage, FPG, Fasting plasma glucose, 2-Hpg, 2-h plasma glucose, HbA1C, Glycosylated hemoglobin

## Abstract

**Purpose:**

To explore differences in the changes of clinical and CT manifestations related to liver abscess before and after CT-guided interventional therapy between patients with and without Diabetes Mellitus (DM).

**Materials and methods:**

Fifty-eight consecutive patients with liver abscesses were retrospectively enrolled in this study. All patients underwent upper abdominal contrast-enhanced CT scans before and after CT-guided interventional therapy. They were divided into two groups including the DM group (n=30) and the Non-DM group (n=28) if the liver abscess occurred in patients with and without DM, respectively. The changes in the clinical and CT manifestations related to liver abscess after CT-guided interventional therapy in both groups were statistically analyzed.

**Results:**

After CT-guided interventional therapy, the length of hospital stay, white blood cell recovery time and drainage tube removal time in the DM group were longer than in the Non-DM group (all p-values < 0.05). The incidence of postoperative complications in the DM group was higher than in the Non-DM group (p < 0.05). As shown on CT, the postoperative reduced percentage of maximum diameter of abscess cavity and the reduction rate of edema band surrounding the liver abscess in the DM group were smaller than in the Non-DM group (both p-values < 0.05). The time intervals of the previous characteristic changes on CT before and after interventional therapy in the DM group were longer than in the Non-DM group (all p-values < 0.05).

**Conclusions:**

The liver abscesses patients with DM could not have a faster recovery and better therapeutic effect than those without DM after the CT-guided interventional therapy.

## Highlights

• Liver abscess patients with diabetes mellitus have featured presentations.
• Liver abscess patients with diabetes mellitus have featured changes after therapy.
• Liver abscess patients with diabetes mellitus cannot have a better therapeutic effect.

## Introduction

Liver Abscess (LA) is a common clinical problem and is caused most commonly by pyogenic, amoebic, or mixed infections.^1^ As a serious infectious disease, LA has shown a trend of high mortality.^1^^,^^2^ LA can be related to some diseases that may lead to mortality.^2^ The diabetes Mellitus (DM) prevalence is relatively high among LA patients and can be up to 35.3%.^3^ In addition, DM status may be apt to result in recurrent infection and severe complications in LA patients.^4^ Meanwhile, poor control of blood glucose may aggravate the situation.^5^ The authors can presume that the presentations after treatments of DM-related LA can be different when compared with LA patients without DM.

In the past, surgical drainage was the traditional mode of treatment in patients with LA.^6^ However, surgical drainage was associated with remarkably high morbidity and mortality rates (10%–47%).^7^ Over the last two decades, outcomes in patients presenting with LA have improved as a result of advances in radiological diagnosis and percutaneous treatment options.^8^, ^9^, ^10^ Currently, patients are treated with antibiotics along with CT-guided Percutaneous Needle Aspiration (PNA) and CT-guided Percutaneous Catheter Drainage (PCD), and surgical drainage is used only in patients who fail to respond to the previous treatments.^11^ Previous studies have shown that both PNA and PCD can be effective and safe.^12^

As far as the authors know, whether differences exist between LA patients with and without DM after CT-guided interventional therapy is still unknown. Therefore, the purpose of this investigation was to explore the differences in the changes in clinical and CT manifestations related to liver abscess before and after CT-guided interventional therapy between patients with and without DM for timely precise treatment decision-making.

## Materials and methods

### Ethics statement

This retrospective study was approved by the present hospital's institutional ethics committee (Approval No. 2020-9-57), and all patients signed written informed consent before the study.

### Study participants

From January 2020 to June 2021, 63 consecutive hospitalized patients, who were diagnosed with LA according to the International Classification of Disease (Clinical Modification 572.0) and treated at the present study's hospital, were enrolled in this study. The diagnosis of LA was based on the clinical features, imaging and laboratory results including blood and *pus* cultures. Patients were required to meet the following inclusion criteria: (1) The patients were presented with clinical features such as chills, fever, abdominal pain and fullness; (2) The laboratory testing showed abnormal results such as abnormal white blood cell, and/or abnormal liver function; (3) The abscess was diagnosed by an etiological test of the blood; (4) Abdominal ultrasonography, Computed Tomography (CT), or magnetic resonance imaging depicted the abscess cavity in the liver; and (5) CT-guided interventional therapy (PNA or PCD) was performed without surgical treatment. The exclusion criteria were as follows: (1) The patients did not have clear clinical or image records (n=2), or (2) The patients did not receive the complete treatment (n=3). Ultimately, 58 patients were enrolled in the present study. The enrolled patients were divided into two groups including the DM group (n=30) and the Non-DM group (n=28) if the liver abscess occurred in patients with and without DM, respectively. As for the DM group, the criteria for type II DM were defined according to the 2017 standards:^13^ (1) Fasting Plasma Glucose (FPG) ≥ 126 mg/dL (7.0 mmoL/L); (2) 2h Plasma Glucose (2-hPG) ≥200 mg/dL (11.1 mmoL/L); (3) Glycosylated Hemoglobin (HbA1C) ≥6.5% (48 mmoL/moL); (4) A random plasma glucose ≥200 mg/dL (11.1 mmoL/L) in patients with classic symptoms of hyperglycemia or hyperglycaemic crisis. All enrolled patients underwent upper abdominal contrast-enhanced CT scans one week before and one to two weeks after the CT-guided interventional therapy.

### CT-guided interventional therapy

The CT-guided interventional therapeutic instruments were a disposable anesthesia puncture kit, 22G liver puncture needle, guiding wire, dilator external drainage tube, connecting bag, surgical blade, needle and suture, etc. 2% lidocaine, antibiotic, saline and nonionic contrast media were used for CT-guided interventional therapy.

During CT-guided interventional therapy, the patients with LA in both groups were placed in the supine position and received a conventional unenhanced CT scan, and the needle direction and distance for this treatment were measured on the CT data. The puncture point was determined, and the body surface was underlined with a bold black pen. The shop towel on each patient was conventionally disinfected after anesthesia. When the puncture through the puncture point was performed with a 22G needle, the patients were asked to be breathless avoiding rough cough and deep breathing to keep the needle staying in the required depth. The *pus* bacteria culture was withdrawn until 20 mL *pus* was drawn out. Subsequently, a contrast-enhanced CT scan was carried out after the injection of the contrast agent into an antecubital vein to show the morphology of the *pus* cavity. And then, a guide wire was placed into the *pus* cavity via the 22G needle, the needle was pulled out, and a small mouth was cut in the local skin with a blade. An expander was then inserted into the puncture site along the guiding wire, and the puncture site was expanded repeatedly several times till the expander was pulled out. The external drainage tube was inserted into the abscess cavity by using Seldinger technology at the lower part of the abscess cavity, which was confirmed by another CT scan. The previous small mouth at the puncture point in the local skin was sutured with several needles, and the drainage tube was fixed and connected with a tee tube connecting a drainage bag. The drainage tube was repeatedly rinsed with sterile saline 24 hours after the interventional therapy, once every 3 days. The drainage tube was removed when the abscess cavity diameter was less than 1 cm, no *pus* was extracted, and the patient's general condition was well. The selection of antibiotics after the interventional therapy, mainly focusing on the selection of antibiotics against aerobic bacteria and anaerobic bacteria, is based on the results of *pus* culture and drug sensitivity.

The patients with LA in both groups underwent upper abdominal contrast-enhanced CT scans with two 64 multidetector scanners (LightSpeed VCT, GE Medical systems, USA) one week before CT-guided interventional therapy, and during the follow-up period (one to two weeks) after the previous treatments till no *pus* drainage. The CT scanning parameters in each patient were 120 kVp of peak voltage, 200 mA of tube current (automatic exposure control employed), rotation time of 0.5 sec, collimation of 64 × 0.6 mm, the pitch of 0.9, slice thickness of 5 mm, and matrix of 512 × 512 mm.

### Data collection

Data were collected by reviewing the electrical medical records of each patient. The records included demographic characteristics (age and sex), blood glucose levels, hospital stay, laboratory values, CT presentations, and postoperative complications including hemorrhage, bile leakage, infection of the incision, and peritonitis.

### Statistical analysis

SPSS version 23.0 software (IBM Corporation, Armonk, NY, USA) was used to analyze the data. For continuous variables, the study was expressed as mean ± standard deviation whereas the authors used frequency and percentage for categorical variables. The authors applied the Student's *t*-test for continuous variables, and the Chi-Squared test or Fisher's exact test for categorical data. A p-value < 0.05 was considered statistically significant.

## Results

### Changes in demographic characteristics

In the cohort of a total of 58 enrolled patients with LA, the demographic characteristics are listed in Table 1. The mean glycemia was significantly higher in the DM group than in the Non-DM group (16.43 ± 5.62 mmoL/L vs. 5.44 ± 1.09 mmoL/L, p = 0.001). Between the DM group (n = 30) and the Non-DM group (n = 28), there were no differences in gender and age (p-values = 0.825 and 0.596, respectively).

**Table 1:** Characteristics of clinical and CT findings between liver abscess patients with (DM) and without (Non-DM) diabetes mellitus.

Characteristic	DM group	Non-DM group	p
Clinical Features			
Age (year)	55.96±13.51	57.73±11.72	0.596
Sex (%)			0.825
Male	18 (60.0)	16 (57.1)	
Female	12 (40.0)	12 (42.9)	
Glycaemia (mmoL/L)	16.43±5.62	5.44±1.09	0.001
Hospitalization day (day)	27.52±12.29	13.87±9.36	0.005
WBC recovery time (day)	4.90±1.24	4.25±1.14	0.043
Drainage tube removal time (day)	12.06±3.45	9.45±2.38	0.022
Postoperative complications (%)	16 (53.3)	5 (17.9)	0.005
Hemorrhage	5 (31.3)	2 (40.0)	
Bile leakage	4 (25.0)	2 (40.0)	
Infection of incision	5 (31.2)	1 (20.0)	
Peritonitis	2 (12.5)	0	
CT Features			
Maximum diameter of preoperative abscess cavity (cm)	7.82±1.99	5.85±2.30	0.001
Postoperative reduced percentage of maximum diameter (%)	39.7	75.2	<0.001
Reduction percentage of >50% edema band surrounding abscess (%)	30.0	67.9	<0.001
Time interval of image feature changes (day)	5.57±1.22	3.11±1.50	<0.001

Notes: WBC, White Blood Cell. For continuous variables, the results are expressed as mean ± standard deviation; and frequency and percentage are used to describe the categorical variables.

After the CT-guided interventional therapy, the hospitalization days in the DM group were longer than in the Non-DM group (p = 0.005). The white blood cell recovery time in the DM group was longer than in the Non-DM group (p = 0.043). The drainage tube removal time in the DM group was longer than in the Non-DM group (p = 0.022). The incidence of postoperative complications in the DM group was higher than in the Non-DM group (p = 0.005).

### Changes in CT presentations after interventional therapy

**Fig. 1:**
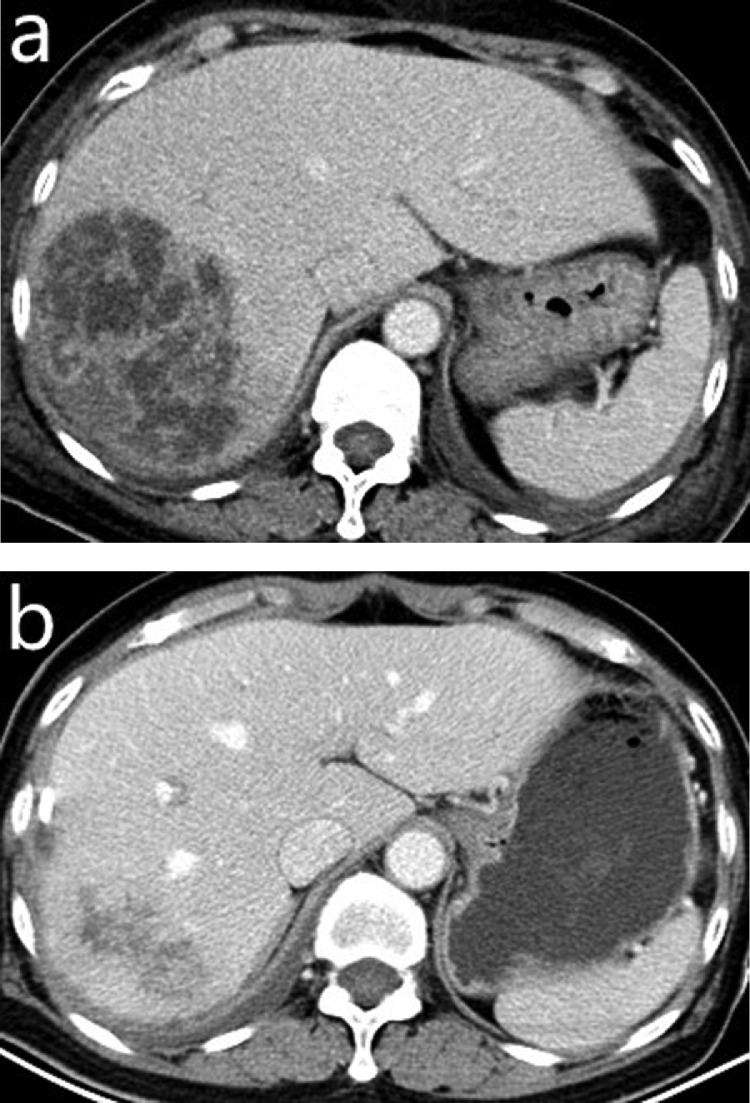
In a 56-year-old liver abscess male with diabetes mellitus, the contrast-enhanced portal-venous phase CT images show the preoperative liver abscess cavity (a), and the obvious shrinkage of it (b) and no *pus* can be drained 32 days after the CT-guided interventional therapy.

**Fig. 2:**
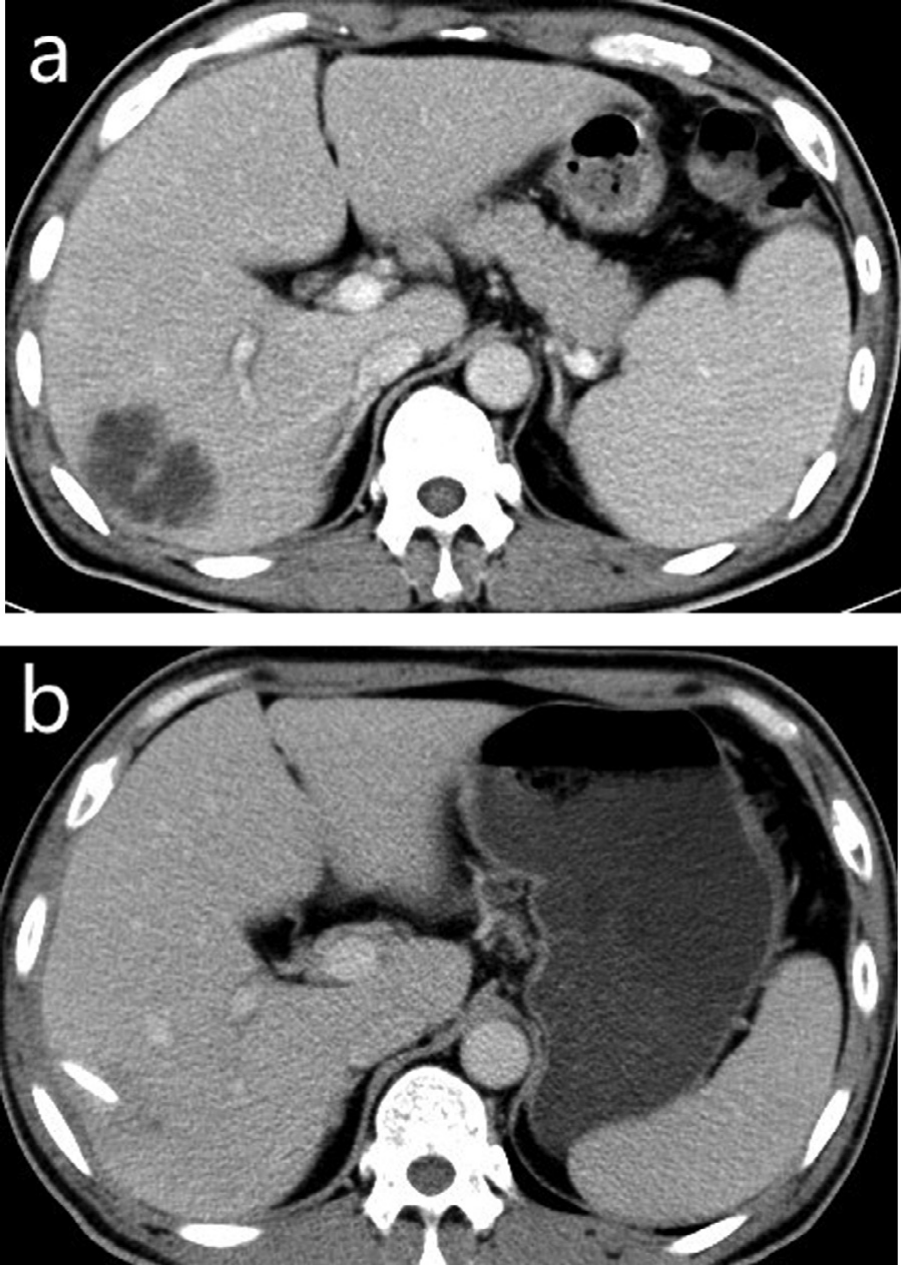
In a 53-year-old liver abscess male without diabetes mellitus, the contrast-enhanced portal-venous phase CT images depict the preoperative liver abscess cavity (a) and the shrinkage of it (b), and no pus drainage can be found 18 days after the CT-guided interventional therapy.

As listed in Table 1, the postoperative reduced percentage of maximum diameter of the abscess cavity after CT-guided interventional therapy in the DM group was smaller than that in the Non-DM group (p < 0.001) (Figs. 1 and 2). The reduction rate of the edema band surrounding the liver abscess in the DM group was smaller than that in the Non-DM group (p < 0.001). The time interval of the previous characteristic changes on CT before and after the interventional therapy in the DM group was longer than that in the Non-DM group (all p-values < 0.05).

## Discussion

LA can be related to some diseases such as DM, nonalcoholic fatty liver disease, hepatocellular carcinoma, et al.^14^^,^^15^ The increasing incidence and high mortality of DM patients with LA have become an important health problem in the hepatobiliary system that plagues humans.^4^ DM is a risk factor for LA with a hazard risk rate of 3.6 to 9-fold,^16^^,^^17^ and LA can directly lead to liver damage, abnormal bile secretion, and increasing portal vein infection.^18^^,^^19^ Autoimmunity in patients with DM was damaged, and their responses to invading microorganisms, including the bactericidal effect of phagocytes and neutralization of chemical toxins, serum opsonin, and cellular immunity, could be easily inhibited, which can weaken the ability of the liver to clear bacteria, making the bacteria easy to colonize and multiply to form liver abscesses.^20^

LA and DM are high-wasting diseases, and often progress rapidly when both occur. Meanwhile, the development of LA has unique characteristics, and the mortality rate is high during the onset period.^21^ DM patients with LA could have a significant impact on overall survival, especially in the short onset period. Researchers think that the physique of DM patients tends to be fragile when suffering from LA, and the condition will be more complicated and the mortality will be higher.^17^ They found that the LA patients with and without DM had a mortality rate of 24.8% and 18.0% within 30 days after discharge, respectively.^17^

In the modern era of minimal invasiveness, image-guided percutaneous treatment (either needle aspiration or catheter drainage) can bridge the gap between operative treatment and conservative with a minimally invasive procedure and has replaced surgery as the first-line treatment for liver abscess.^22^ The purpose of this study was to compare the changes in clinical and CT characteristics related to LA before and after CT-guided interventional therapy between patients with and without DM so as to improve the therapeutic effects of LA patients.

As is known, patients with DM are generally in an immunocompromised state. DM has been shown to be associated with unfavorable outcomes among patients with LA in previous studies.^23^^,^^24^ The present clinical findings supported the previously reported findings. LA patients with DM could not have a faster recovery and better therapeutic effect after CT-guided interventional therapy than without DM. The present findings indicate that the administration of LA patients with DM is generally more complex than that of LA patients without DM. After CT-guided interventional therapy, the hospitalization days in the DM group were longer than in the Non-DM group (27.52 ± 12.29 d vs. 13.87 ± 9.36 d). The white blood cell recovery time in the DM group was longer than in the Non-DM group (4.90 ± 1.24 d vs. 4.25 ± 1.14 d). The drainage tube removal time in the DM group was longer than in the Non-DM group (12.06 ± 3.45 d vs. 9.45 ± 2.38 d). The incidences of postoperative complications such as hemorrhage, bile leakage, infection of the incision, and peritonitis in the DM group were higher than in the Non-DM group (53.3% vs. 17.9%). The clinical features of LA patients with DM can be explained as follows. Hyperglycemia in tissues can easily lead to hypertonic cells and weakened the regenerative repair function, which may delay puncture healing and lead to more complications.^25^ As a result of the weakened immune system, it takes a longer time for white blood cells to return to normal, resulting in longer hospital stays.

Clinically, the CT findings also reveal that LA patients with DM could not have a faster recovery and better therapeutic effect after CT-guided interventional therapy than without DM. The authors found that the maximum diameter of preoperative abscess cavity in LA patients with DM was larger than in LA patients without DM and that the postoperative reduced percentage of maximum diameter of abscess cavity in LA patients with DM was smaller than in LA patients without DM (39.7% vs. 75.2%). Although the present findings were similar to the published report that the diameter of abscesses in the DM group could be generally larger,^26^ for the first time, the authors found that the postoperative reduced percentage of maximum diameter of abscess cavities in LA patients with DM smaller than in LA patients without DM. In addition, the authors also reported that the reduction rate of the edema band surrounding the liver abscess in the DM group was smaller than in the Non-DM group (30.0% vs. 67.9%). The time interval of the previous changes in image characteristics after the interventional therapy in the DM group was longer than in the Non-DM group (5.57 ± 1.22 d vs. 3.11 ± 1.50 d). The imaging monitoring showed that the recovery time of LA patients with DM after the interventional therapy was longer than that of LA patients without DM (5d vs. 3d), which may be due to the decreased and vulnerable immune system in the DM patients.

The limitations of this study mainly focus on its relatively small sample size and single-centered traits. However, the findings the authors have gained can be a helpful reference for clinicians and researchers to further enrich their experience of diagnosis and treatment of LA patients with DM. In addition, the microbiological analysis of the contents of the liver abscess was not compared between the patients with and without basic diabetes mellitus. The authors will perform the relevant study in the future.

In conclusion, liver abscess patients with diabetes mellitus have characteristic changes in clinical and CT manifestations before and after CT-guided interventional therapy when compared with liver abscess patients without diabetes mellitus. After CT-guided interventional therapy, liver abscess patients with diabetes mellitus could not have a faster recovery and better therapeutic effects than those without diabetes mellitus. The present findings indicate that diabetes mellitus is an important risk factor for liver abscess patients, and effective management of blood glucose levels should be recommended besides the treatment of a liver abscess.

## Authors’ contributions

Conceptualization: Yulin He; Data curation: Yuxiang Chen, Xunfu Lai, Yuping Zhu, and Mengting Wang; Formal analysis: Yuxiang Chen; Investigation: Yuxiang Chen; Methodology: Yulin He; Project administration: Yulin He; Resources: Yulin He; Software: Yulin He; Supervision: Yulin He; Validation: Yuxiang Chen, Xunfu Lai, Yuping Zhu, and Mengting Wang; Visualization: Yuxiang Chen; Roles/Writing ‒ original draft: Yuxiang Chen, and Yulin He; Writing ‒ review & editing: Yuxiang Chen, and Yulin He.

## Conflicts of interest

The authors declare no conflicts of interest.
